# Organo-Selenium-Containing Polyester Bandage Inhibits Bacterial Biofilm Growth on the Bandage and in the Wound

**DOI:** 10.3390/biomedicines8030062

**Published:** 2020-03-17

**Authors:** Phat Tran, Tyler Enos, Keaton Luth, Abdul Hamood, Coby Ray, Kelly Mitchell, Ted W. Reid

**Affiliations:** 1 Department of Ophthalmology & Visual Sciences, TTUHSC, Lubbock, TX 79430, USA; Phat.tran@ttuhsc.edu (P.T.); tyler.enos@phhs.org (T.E.); keaton.luth@gmail.com (K.L.); Coby.ray@ttuhsc.edu (C.R.); Kelly.mitchell@ttuhsc.edu (K.M.); 2 Department of Microbiology & Immunology, TTUHSC, Lubbock, TX 79430, USA; abdul.hamood@ttuhsc.edu; 3 SelenBio, Inc., Austin, TX 78735, USA

**Keywords:** anti-biofilm formation, wound healing, organo-selenium, dressing technology

## Abstract

The dressing material of a wound plays a key role since bacteria can live in the bandage and keep re-infecting the wound, thus a bandage is needed that blocks biofilm in the bandage. Using an in vivo wound biofilm model, we examined the effectiveness of an organo-selenium (OS)-coated polyester dressing to inhibit the growth of bacteria in a wound. *Staphylococcus aureus* (as well as MRSA, Methicillin resistant *Staph aureus*), *Stenotrophomonas maltophilia*, *Enterococcus faecalis*, *Staphylococcus epidermidis*, and *Pseudomonas aeruginosa* were chosen for the wound infection study. All the bacteria were enumerated in the wound dressing and in the wound tissue under the dressing. Using colony-forming unit (CFU) assays, over 7 logs of inhibition (100%) was found for all the bacterial strains on the material of the OS-coated wound dressing and in the tissue under that dressing. Confocal laser scanning microscopy along with IVIS spectrum in vivo imaging confirmed the CFU results. Thus, the dressing acts as a reservoir for a biofilm, which causes wound infection. The same results were obtained after soaking the dressing in PBS at 37 °C for three months before use. These results suggest that an OS coating on polyester dressing is both effective and durable in blocking wound infection.

## 1. Introduction

Organo-selenium, unlike other biocidal agents, such as silver ions, can be covalently attached to various materials with no loss of catalytic activity. Previous studies have shown that selenium (Se) covalently bound to a solid matrix retains its ability to catalyze the formation of superoxide radicals (O_2_^·–^) in a continuous process in which Se donates an electron to oxygen and acquires one from sulfur compounds (such as glutathione) that are present in all body fluids [1]. Recently, it has been shown that organo-selenium can be attached to different biomaterials and medical devices to block the formation of *P. aeruginosa* and *S. aureus* biofilms on those materials [2,3,4,5,6]. Once a biofilm is formed on one of these devices, the only remedy is to replace the infected device, making prevention paramount. Selenium has additionally shown promise for this purpose in that unlike other antibacterial agents, longer periods of exposure to an aqueous environment do not compromise the effectiveness of the coating [2,3,4,5,6].

Biofilm formation occurring in a wound itself inhibits wound healing [7,8]. Antibiotics or silver embedded in gauze have been shown to be efficacious in preventing wound infection [9]. However, there are several problems associated with these treatments. One is the increasing number of antibiotic-resistant bacteria, including *P. aeruginosa* and *S. aureus*, which severely limits the choices for antibiotic treatment. A second is that in order to be effective, silver compounds must leach into the surrounding tissue, which can cause tissue damage [9]. Silver also discolors the wound, which makes it hard to assess wound healing. Because of these problems it is essential to investigate alternative antibacterial agents that can contact the infected tissues and prevent the development of bacterial biofilms. In this study, we examined the effectiveness of a selenium-coated polyester for inhibiting the growth of different bacteria. Polyester is used in medicine primarily as a bandage material. The surface is susceptible to biofilm formation, which can cause an infection of the wound. The idea of this study is not only to prevent biofilm formation in the bandage but to lessen the chance of biofilm formation in the wound.

## 2. Experimental Section

### 2.1. Coating Polyester Dressing with Polymerized Se-AAEMA

The Se-AAEMA [seleno-2 (methacryloyloxy)ethyl acetoacetate] monomer was purchased from Eburon Organics International (catalog No. 700.010; Lubbock, TX, USA). Se-AAEMA (22% [wt/wt] selenium) stock solution was dissolved and diluted in 99.99% 2-(methacryloyloxy)-ethyl acetoacetate (AAEMA) to yield the 1% Se-AAEMA (wt/wt), used for the coating. Then, 100% ivory polyester fabric purchased from a local Walmart store (#M30-23) was cut into 4 inch x 4 inch square pieces and soaked in sterilized distilled water overnight. The washed polyester dressing was then dried at 37 °C in an oven and coated with AAEMA or Se-AAEMA. Briefly, the dressings were soaked in AAEMA or Se-AAEMA for 30 min. Excess AAEMA or Se-AAEMA was removed from the dressings by passing the soaked bandage through a roller press (mechanical cloth wringer). The AAEMA or Se-AAEMA polyester dressings were then sprayed with H_2_O_2_ using a bottle sprayer to initiate free radical polymerization. This has little or no effect on the bandage material. As a result of the in situ polymerization, the polymer is held in place by a combination of van der Waals forces and physical interlinking with the dressing material. The dressings were then transferred to a 66 °C curing oven. After curing, the dressings were washed twice (1 h each) in sterile phosphate-buffered saline (PBS, pH 7.4) at 37 °C. The dressings were then dried, cut into 1 cm × 1 cm and 1.5 cm × 1.5 cm pieces, sterilized by dried autoclaving, dried again, and stored at room temperature until being used in the assays. All percentages listed for the different concentrations of coating are the percentages of selenium in the coating.

### 2.2. Bacterial Strains

All strains were grown in Luria Bertani (LB) Broth, Mueller Hinton Broth, or on LB Agar plates at 37 °C. *S. aureus* AH133 (TTUHSC laboratory strain) was used. This is a lab strain that constitutively expresses green fluorescent protein from a plasmid (pCM11) in the presence of 1 μg/mL erythromycin [10]. *Pseudomonas aeruginosa* strain PAO1/pMRP9-1was also grown. This strain carries the plasmid pMRP9-1 that contains the gene that codes for the green fluorescent protein [11]. To maintain the plasmid, the strain was grown in the presence of 300 μg/mL carbenicillin. The Methicillin-resistant *S. aureus* and *S. epidermidis* clinical isolates were obtained from leg wounds or abscess wounds from the Clinical lab at Texas Tech University Health Sciences Center under an approved Institutional Review Board protocol of the Texas Tech University Medical Center in Lubbock, Texas. *Staphylococcus aureus* Lux Xen29 was also used, a strain with a plasmid containing a gene that codes for the luciferase protein [12]. To maintain the plasmid, the strain was grown in the presence of 40 µg/mL kanamycin. This strain was available at our lab at Texas Tech University Health Sciences Center. *Pseudomonas aeruginosa* PAO1 Lux Xen5 strain was available at our lab at Texas Tech University Health Sciences Center [12]. Finally, *Stenotrophomonas maltophilia* ATCC^®^ 53199™ was available at our lab at Texas Tech University Health Sciences Center. All bacterial stock was stored at −80 °C.

### 2.3. Colony-Forming Unit (CFU) Assay In Vitro

Bacteria were grown overnight, and washed once with PBS (pH 7.4). They were then re-suspended in PBS (pH 7.4) to an optical density (OD_600_) of 0.1 (10^7^ CFU/mL), and serially diluted 10-fold. All PBS assay solutions contained 150 μM reduced glutathione (Sigma Chemical, St. Louis, MO, USA). Ten microliter aliquots containing 10^2^ to 10^3^ colony-forming units were added to the bandages. They were a stack (3 pieces of one square centimeter) containing either AAEMA (control treated with AAEMA with no selenium—listed in the figures as untreated dressing) polyester dressing, or Se-AAEMA test polyester dressing. After the bacterial inoculum was absorbed into the dressings, they were placed on LB agar plates, and the plates were incubated at 37 °C for 24 h. Biofilms were then quantified by determining the CFU per square centimeter of dressing by the following procedure. After incubation, each stack of dressing was rinsed with sterile PBS and then was transferred to a sterile 1.5-mL microcentrifuge tube containing 1 mL of PBS (pH = 7.4) for enumeration of bacteria. The tubes were placed in a water bath sonicator for 5-10 min to loosen the cells within the dressing and then vigorously vortexed 3 times for 1 min to detach the cells. Suspended cells were serially diluted (10-fold) in PBS, and 10-μL aliquots of each dilution were spotted onto LB Agar plates. In addition, the remaining 900-μL zero dilution sample was plated on a different LB Agar plate. Thus, the equation for back calculating the bacterial concentration was CFU × dilution factor × 100, with the exception of the 900-μL sample, which was calculated as CFU × 1. This means that if only one bacterium was originally in the tube, we would have a 90% chance of detecting it. All experiments were done in triplicate, and all measurements were repeated three times.

### 2.4. Mouse Wound Infection Model

Six adult female Balb C mice (including a control group) weighing 20–24 g were anesthetized using a mixture of isoflurane and oxygen, and their backs were shaved. Shaved areas were completely cleansed with 95% ethanol. A flap of skin was lifted and a 1.0-cm^2^ piece of skin was removed centrally in the shaved area with sterile scissors. Either an AAEMA control or Se-AAEMA dressing (1.5 cm × 1.5 cm) was placed on the wound. The dressings were secured in place by applying a clear OPSITE dressing over the back of the mouse. Aliquots containing 10^2^ to 10^3^ CFU of bacteria in 20 μL were injected in the area between the bandage and the wound. The mice were monitored twice a day for signs of infection or distress. The dressings were removed, and the connective tissue around the wound and the tissue under the wound (down to the spinal cord) was then dissected and removed. The extracted dressings and all the dissected tissue were gently rinsed in PBS and homogenized in PBS. Excess PBS was drained from the dressing by touching it to a sterile filter paper and the dressing was then transferred to a sterile homogenized tube containing 2 mL of PBS for enumeration of bacteria by either determination of CFU or by confocal microscopy. All experiments were done at least in triplicate.

Tissue: At the end of the experiment (5 days), the mouse was euthanized (with CO_2_ in a closed chamber) and the connective tissue around the wound and the tissue under the wound (down to the spinal cord) was then dissected and removed. This was then homogenized and used for a CFU assay in a manner similar to that used for the dressing above.

Animals were treated in accordance with the protocol approved by the Institutional Animal Care and Use Committee at Texas Tech University Health Sciences Center in Lubbock, TX, USA.

### 2.5. Biofilm Detection by Assay Using the Dressing

Biofilms were quantified by determining the CFU recovered from the dressings. As above, after removal from the mouse, each piece of dressing was gently washed twice with sterile 1× PBS to remove any planktonic bacteria. Excess PBS was drained from the dressing by using sterile filter paper and the dressing was then transferred to a sterile homogenized tube containing 2 mL of sterile 1× PBS (pH 7.4). The dressings were homogenized to loosen the cells within the biofilm and then vigorously vortexed 3 times for 1 min to detach the cells. Suspended cells were serially diluted (10-fold) in sterile 1× PBS (pH 7.4), and 10-μL aliquots of each dilution were spotted onto LB Agar plates. Bacterial counts were determined as described above. The results were reported as attached CFU per dressings recovered from each mouse. All experiments were done in triplicate.

### 2.6. Biofilm Detection Assay for Tissue

At the end of the in vivo experiment (5 days), the mice were euthanized, and the tissue of the wounds was removed. The tissue was weighed, and it was then homogenized. The CFU assay was performed in a manner similar to the assay used for the dressing above. The results were reported as attached CFU per gram of tissue. All experiments were done in at least triplicate.

### 2.7. Biofilm Analysis by Fluorescence Microscopy

The pieces of polyester dressings at the end of the in vivo 5-day incubation were assayed as described above for the CFU assays. AAEMA control and Se-AAEMA polyester dressing segments from three mice of each group were also examined for the presence of biofilm by confocal laser scanning microscopy (CLSM) using an Olympus Fluoview FV300 (Olympus America, Center Valley, PA, USA). Animals were treated in accordance with the protocol approved by the Institutional Animal Care and Use Committee at Texas Tech University Health Sciences Center in Lubbock, TX, USA.

### 2.8. In Vivo Live Imaging Studies

Mice were also infected with either *Staphylococcus aureus* Lux Xen29 or *Pseudomonas aeruginosa* Lux Xen5 and observed at day 5 after treatment. The mice were lightly anesthetized, and the infected wounds were visualized using an IVIS Lumina XR system with Living Image software (Perkin Elmer, US). This instrument allowed us to directly detect pathogenic infections in living animals by producing images through bioluminescence. After imaging, the mice were euthanized, and the gauze dressing and wound tissues were recovered. The samples were then analyzed by the CFU assay as described above. Imaging experiments were conducted at the Texas Tech University Health Sciences Center Image Analysis Core Facility (Lubbock, TX, USA). All experiments were done in triplicate.

### 2.9. Analysis of the Long-Term Stability of the Organo-Selenium-Containing Polyester Dressing

One square-centimeter strips of the organo-selenium-containing polyester dressing were completely immersed in 1.0 mL of PBS in sterile glass tubes and incubated at 37 °C for a month. At the end of the incubation period, the pieces were removed, dried, sterilized by dried autoclaving, and utilized in the in vitro biofilm assay, as described above, for determination of the durability of the anti-biofilm activity of the coating.

### 2.10. Statistical Analysis

Results of the CFU assays were analyzed with Prism^®^ version 4.03 (GraphPad Software, San Diego, CA, USA) with 95% confidence intervals (CIs) of the difference. Comparisons of the in vivo biofilms formed on Se-free and Se-AAEMA polyester dressings were analyzed by a two-tailed unpaired t-test to determine significant differences. All experiments were done at least in triplicate.

## 3. Results

### 3.1. In Vitro Testing of the Selenium-Coated Polyester Bandage against Biofim Formation by Different Bacterial Strains

**Figure 1:**
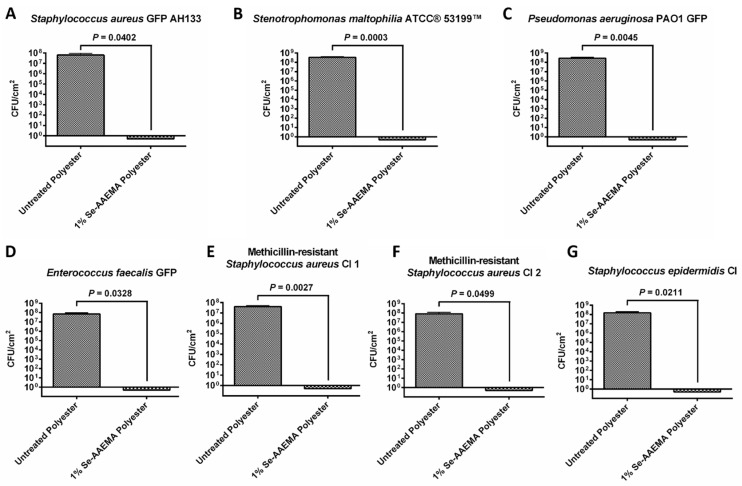
Graph of the colony-forming units of (**A**) *Staphylococcus aureus* GFP AH133, (**B**) *Stenotrophomonas maltophilia* ATCC^®^ 53199™, (**C**) *Pseudomonas aeruginosa* PAO1 GFP, (**D**) *Enterococcus faecalis* GFP, (**E**) Methicillin-resistant *Staphylococcus aureus* CI 1, (**F**) Methicillin-resistant *Staphylococcus aureus* CI 2, and (**G**) *Staphylococcus epidermidis* CI biofilms formed on untreated polyester and 1% Se-AAEMA polyester. Values represent the means of triplicate experiments ± SEM. A two-tailed unpaired t test was used to determine statistical significance. Untreated polyester has AAEMA but no selenium.

For the bandages coated with organo-selenium in the form of 1% Se-AAEMA (see Figure 1), after 24 h of growth, between 7–8 logs of inhibition were seen (by CFU assay) for all the different bacterial strains (*Stenotrophomonas maltophilia*, *Enterococcus faecalis*, *Staphylococcus epidermidis* and *Pseudomonas aeruginosa*), when compared with the control bandage coated with AAEMA containing no selenium. This result was true for all bacteria, whether Gram-negative or Gram-positive.

### 3.2. In Vitro Visualization of Biofilm Formation by Confocal Laser Scanning Microscopy for Different Bacteria on a Polyester Bandage with and without a Selenium Coating

**Figure 2:**
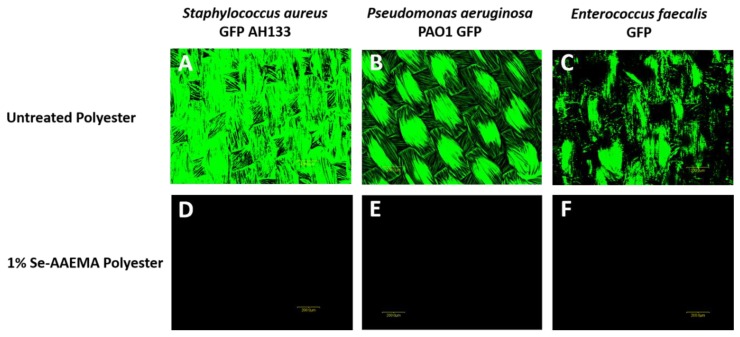
In Vitro study. Representative confocal laser scanning microscopy images of (**A**) *Staphylococcus aureus* GFP AH133, (**B**) *Pseudomonas aeruginosa* PAO1 GFP, and (**C**) *Enterococcus faecalis GFP* biofilm formed on untreated polyester and 1% Se-AAEMA polyester. Untreated polyester has AAEMA but no selenium. (**D**–**F**) are the same samples as those above however they have Se-AAEMA. As seen all the bacteria are eliminated. The bar is 200 μm.

As can be seen in Figure 2, biofilms were formed for 24 h on the polyester that was only coated with AAEMA, while no biofilm was formed on the polyester with the selenium-AAEMA. The non-selenium-coated fibers were seen to serve as a good binding material for the different bacteria. These results are consistent with those found from the CFU studies seen in Figure 1.

### 3.3. In Vivo Studies of Biofilm Formation on a Polyester Dressing and in the Underlying Wound on a Mouse Wound Model

**Figure 3:**
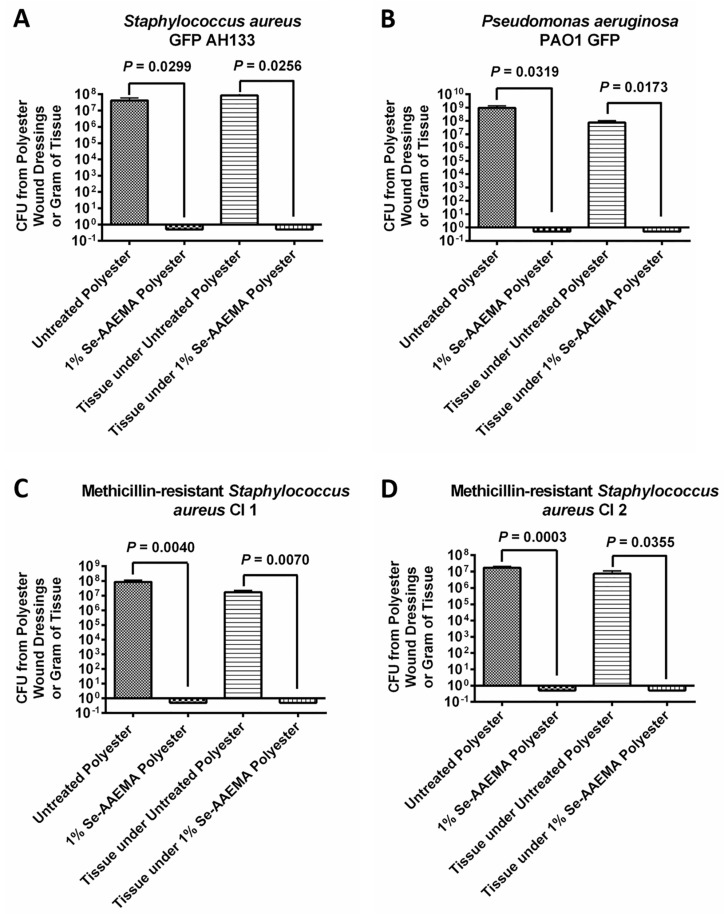
Graph of the colony-forming units of (**A**) *Staphylococcus aureus* GFP AH133, (**B**) *Pseudomonas aeruginosa* PAO1 GFP, (**C**) Methicillin-resistant *Staphylococcus aureus* CI 1, and (**D**) Methicillin-resistant *Staphylococcus aureus* CI 2 biofilms formed on the polyester dressings and in the tissue under the polyester dressings on a mouse wound. Values represent the means of six replicate experiments ± SEM. A two-tailed unpaired t test was used to determine statistical significance. Untreated polyester has AAEMA but no selenium.

**Figure 4:**
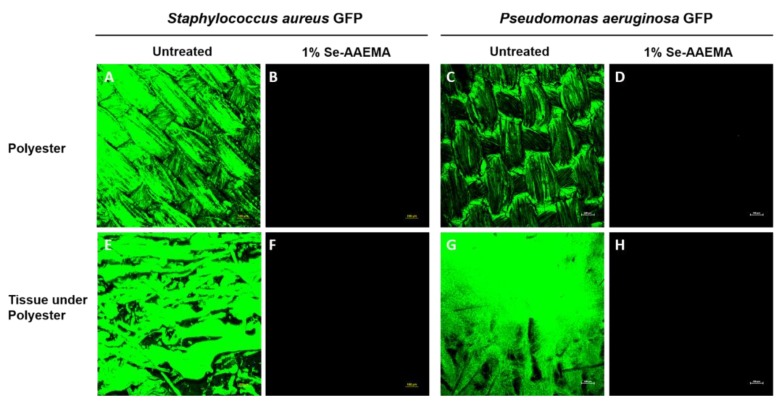
Mouse wounds after 5 days. Representative confocal laser scanning microscopy images of (**A**) *Staphylococcus aureus* GFP AH133 with untreated polyester showing bacteria in the AAEMA bandage, and (**B**) is the same except it is with a Se-AAEMA bandage, while (**E**) is the tissue under bandage (**A**), and (**F**) is the tissue under bandage (**B**). (**C**) is *Pseudomonas aeruginosa* PAO1 GFP biofilms formed on the AAEMA treated polyester dressings and (**D**) is the same as (**A**) except it is with the SeAAEMA treated dressing. (**G**) is the tissue under the polyester dressings (**A**), and (**H**) is the tissue under the SeAAEMA bandage (**D**). Bar is 100 μm.

Different bacteria were injected under a polyester bandage dressing, either with or without a selenium coating, which covered a mouse wound model. These were then allowed to grow for 5 days. As shown in Figure 3, not only did the selenium coating result in between a 7–8 logs reduction (100%) in the amount of biofilm on the dressing compared with the non-selenium containing dressing, but it also produced a 7–8 logs of reduction (100%) in the underlying tissue of the wound. These results were found for *Staphylococcus aureus*, *Pseudomonas aeruginosa*, and two different clinical strains of MRSA. These results are confirmed by confocal laser scanning microscopy in Figure 4.

### 3.4. In Vivo Live Imaging of the Growth of Bacteria in the Wound

**Figure 5:**
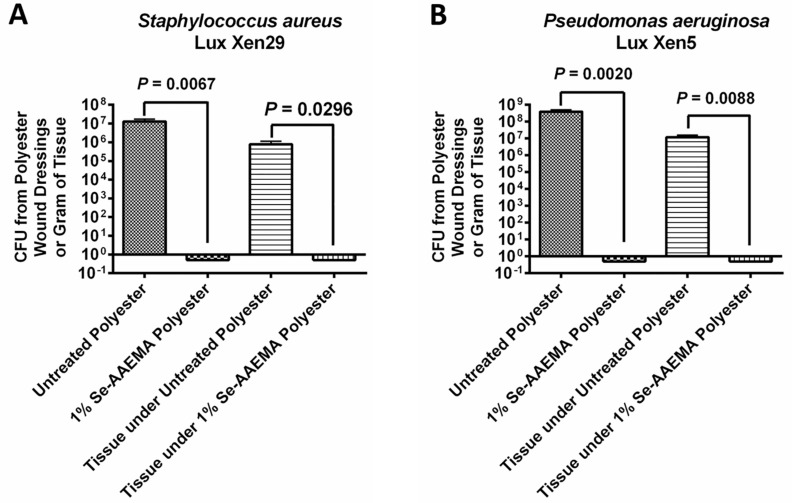
Graphs of the colony-forming units recovered from the effect of AAEMA and SeAAEMA coated polyester dressing (**A**) *S. aureus* Lux Xen29 and (**B**) *P. aeruginosa* Lux Xen5 in the polyester dressings and the mouse wound tissue. Untreated polyester has AAEMA but no selenium.

**Figure 6:**
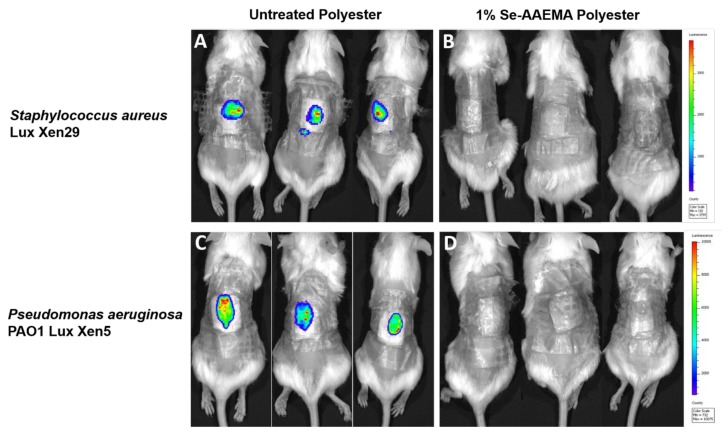
Representative IVIS in vivo live images of (**A**) *S. aureus* Lux Xen29 under AAEMA polyester dressing and (**B**) Se-AAEMA polyester dressing. (**C**) *P. aeruginosa* Lux Xen5 biofilms formed under the AAEMA polyester dressings and (**D**) the Se-AAEMA polyester dressing.

In order to image the growth of bacteria in the wound, bacteria with the Lux gene were used. Mice were infected with either *Staphylococcus aureus* Lux Xen29 or *Pseudomonas aeruginosa* Lux Xen5 under the polyester bandage and observed at day 5 after treatment. The actual growth of bacteria with this gene present is seen in Figure 5 for bacteria in the dressing and in the wound. As shown in the pictures (Figure 6), no active infection could be detected in under the Se-AAEMA polyester dressing while each mouse with a control (AAEMA) bandage demonstrated obvious active wound infection.

### 3.5. Stability of the Selenium-Coated Polyester Bandage in Preventing Biofilm Formation

**Figure 7:**
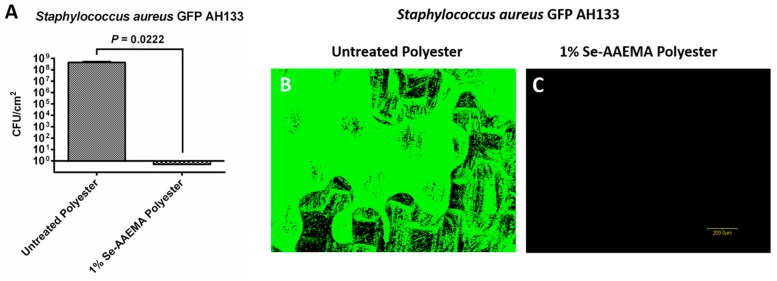
Stability study of bandage after one month in PBS at 37 °C. The inhibitory effect of the organo-selenium coating is long-lasting against *Staphylococcus aureus* GFP AH133 biofilms formed on untreated polyester and 1% Se-AAEMA polyester, which were previously soaked in 1× PBS (pH = 7.4) for three months. Values represent the means of quadruplicate experiments ± SEM. (**A**) two-tailed unpaired t test was used to determine statistical significance. Representative confocal laser scanning microscopy images of (**B**) *Staphylococcus aureus* GFP AH133 biofilms formed on untreated polyester and (**C**) 1% Se-AAEMA polyester, which were previously soaked in 1× PBS (pH = 7.4) for three months. Untreated polyester has AAEMA but no selenium. Bar is 200 m.

A stability study was carried out to see if the organo-selenium-coated bandages retained their biofilm inhibitory activity after one month of storage in PBS solution at 37 °C. As seen in Figure 7, there was still 8 logs (100%) of inhibition present against *S. aureus* GFP AH133 with the 1% Se-AAEMA coating. These results were confirmed by visualization with the confocal laser scanning microscope (Figure 7B,C).

## 4. Discussion

These experiments studied whether selenium atoms that are covalently incorporated into a methacrylate polymer (AAEMA) used to coat a polyester bandage could block bacterial attachment to the bandage and influence biofilm formation in a wound. Selenium is unique amongst the elements in its ability to be attached to an organic molecule with a covalent bond and yet still retain its ability to catalyze the formation of superoxide radicals. This is done by selenium donating an electron to oxygen to form superoxide, resulting in the oxidation of the selenium atom. The oxidized selenium atom can then react with sulfhydryl groups present in all body fluids, such as glutathione, to become reduced, resulting in oxidization of the sulfhydryl (disulfide bond formation). This completes the catalytic cycle by essentially taking an electron from sulfur and giving it to oxygen. The advantage of this mechanism is that the selenium remains unchanged and attached to the polymer yet creates a local layer of superoxide on the surface of the material. However, the half-life of superoxide is short and does not travel far. It is probably due to this short half-life that previous studies have shown that there is no toxic effect upon nearby mammalian cells [2,4].

Based upon the mechanism described above, we expected the selenium coating to block bacterial attachment to the dressing. However, we were interested to find that the coating on the bandage also inhibited biofilm formation in the wound as well, even though the bacteria were injected into the area between the bandage and the wound. Since selenium does not leave the bandage and the half-life of the generated superoxide is very short (milliseconds at best), we expected the selenium mechanism to have more limited effects upon the wound tissue, especially during the 5 days of the experiment. One possible mechanism for the effect of the selenium coating on wound infection is that biofilm formation in a typical wound requires instigation by biofilm formation on the bandage. After the injection of the bacteria, the organisms that initially go into the wound are probably destroyed by the normal host cell defense mechanisms. Those bacteria that invade the control bandage, however, can form a biofilm on the bandage. This biofilm can release planktonic bacteria and toxins over time and continuously re-infect the wound. During this re-infection, toxins will kill cells in the wound, and these dead or compromised cells can serve as a matrix for biofilm formation in the wound itself. However, with the selenium-coated bandage, there is no biofilm formation in the bandage since the coating kills 100% of the bacteria that attempt to attach to the bandage. This serves to protect the wound from re-infection. Thus, a selenium-coated bandage should allow the wound requisite time to heal without biofilm formation. This was also confirmed by bioluminescence, which offers a method for monitoring infections in vivo over a period of several days. It is sensitive and non-invasive and requires fewer animals than conventional methodologies. It showed no infection developing in the selenium bandage or the wound over time.

## 5. Conclusions

Organo-selenium monomers can be polymerized into a coating on polyester fabric, which is suitable for medical wound dressings. Organo-selenium-coated polyester dressings significantly inhibited biofilm formation by all bacteria tested (both Gram-negative and Gram-positive). Of importance was the fact that the selenium coating not only inhibited biofilm formation in the dressing but also inhibited biofilm formation in an inoculated wound over a 5-day period. The organo-selenium coating on the polyester dressing was also found to be stable after long soaking, and the inhibitory effect on bacterial biofilm remained. In future experiments, we plan to test the incorporation of the organo-selenium monomer into the polyester polymer itself rather than just coating it on the surface of the dressing. This would likely make a superior dressing that avoids problems with surface wear, leaching, or abrasion, and would allow for more flexibility in manufacturing.
